# The Central Role of Extracellular Vesicles in the Mechanisms of Thrombosis in COVID-19 Patients With Cancer and Therapeutic Strategies

**DOI:** 10.3389/fcell.2021.792335

**Published:** 2022-01-12

**Authors:** Haijiao Jing, Nan Zuo, Valerie A. Novakovic, Jialan Shi

**Affiliations:** ^1^ Department of Hematology, The First Hospital, Harbin Medical University, Harbin, China; ^2^ Department of Research, VA Boston Healthcare System, Harvard Medical School, Boston, MA, United States; ^3^ Department of Medical Oncology, Dana-Farber Cancer Institute, Harvard Medical School, Boston, MA, United States

**Keywords:** COVID-19, cancer, thrombosis, microparticles, phosphatidylserine, treatment strategy

## Abstract

Cancer patients have increased SARS-CoV-2 susceptibility and are prone to developing severe COVID-19 infections. The incidence of venous thrombosis is approximately 20% in COVID-19 patients with cancer. It has been suggested that thrombus formation has been suggested to correlate with severe clinical manifestations, mortality, and sequelae. In this review, we primarily elaborate on the pathophysiological mechanisms of thrombosis in COVID-19 patients with cancer, emphasize the role of microparticles (MPs) and phosphatidylserine (PS) in coagulation, and propose an antithrombotic strategy. The coagulation mechanisms of COVID-19 and cancer synergistically amplify the coagulation cascade, and collectively promotes pulmonary microvascular occlusion. During systemic coagulation, the virus activates immune cells to release abundant proinflammatory cytokines, referred to as cytokine storm, resulting in the apoptosis of tumor and blood cells and subsequent MPs release. Additionally, we highlight that tumor cells contribute to MPs and coagulation by apoptosis owing to insufficient blood supply. A positive feedback loop of cytokines storm and MPs storm promotes microvascular coagulation storm, leading to microthrombi formation and inadequate blood perfusion. Microthrombi-damaged endothelial cells (ECs), tumor, and blood cells further aggravate the apoptosis of the cells and facilitate MPs storm. PS, especially on MPs, plays a pivotal role in the blood coagulation process, contributing to clot initiation, amplification, and propagation. Since coagulation is a common pathway of COVID-19 and cancer, and associated with mortality, patients would benefit from antithrombotic therapy. The above results lead us to assert that early stage antithrombotic therapy is optimal. This strategy is likely to maintain blood flow patency contributing to viral clearance, attenuating the formation of cytokines and MPs storm, maintaining oxygen saturation, and avoiding the progress of the disease.

## Introduction

Cancer patients are vulnerable to severe acute respiratory syndrome coronavirus 2 (SARS-CoV-2) infection. A recent meta-analysis of global data shows that the prevalence of cancer among COVID-19 patients in Europe is up to 22% (Kong et al., 2021). The demand for invasive mechanical ventilation in cancer patients infected with the virus has increased notably, as these patients are more likely to progress to severe pneumonia, and exhibit more severe pathological alterations and damage than other COVID-19 patients (Dettorre et al., 2021). Even with adequate therapy, the worldwide mortality rate for COVID-19 patients with cancer remains high (25.6%) especially in older patients and those with underlying illnesses (Saini et al., 2020). Thrombotic microangiopathies, arterial and venous thrombosis are the leading causes of increased mortality in patients (Yan et al., 2021). Previous studies have shown that prolonged inflammatory signals characterized by excessive release of inflammatory cytokines may be the immunopathological basis and leads to pulmonary endothelial injury, with infiltration of immune cells, and systemic hypercoagulability (Vora et al., 2021). Although pro-inflammatory cytokines are known to promote the generation of various procoagulant factors, they may be insufficient for the systemic thrombosis seen in some severe COVID-19 patients. Consequently, other factors to facilitate thrombus formation exist between inflammation and coagulation. Recently, articles reporting the involvement of microparticles (MPs, a kind of extracellular vesicles) in coagulation have broadly attracted attention. Platelets (PLTs) are the principal source of MPs in the peripheral blood, and MPs have been implicated in an increased risk of thromboembolic events in a broad range of conditions, including cancer and preeclampsia (Tripisciano et al., 2017). Our previous studies have confirmed MPs contribute to the coagulation cascade in the kidneys of COVID-19 patients (Chen et al., 2020). In several malignant cancers, tumor-derived MPs promote a prothrombotic state. Additionally, the cytokines storm of COVID-19 provides a prerequisite for MPs formation, suggesting that MPs exert an essential role in COVID-19 coagulation in patients with cancer. The procoagulant effect of MPs is achieved primarily through externalized phosphatidylserine (PS). However, articles focusing on PS coagulation are comparatively rare, and the specific mechanisms are still not clarified. Thrombosis correlates with high mortality and severe sequelae in COVID-19. However, current antithrombotic therapies focus only on prophylactic anticoagulation and rarely mention the administration of anti-platelet aggregation drugs and thrombolytic drugs, despite the fact that thromboembolic events are commonly encountered in patients following anticoagulation therapy (Ortega-Paz et al., 2021). A recent article in New England Journal of Medicine reported that patients with severe COVID-19 receiving anticoagulant therapy at a therapeutic dose do not experience a change in coagulant state or decreased mortality (ATTACC Investigators et al., 2021). Therefore, it is crucial that anticoagulation treatment be administered quickly after diagnosis, in the early or middle stages, to ensure the best outcome. High susceptibility to SARS-CoV-2 infection, a propensity to thrombosis, and higher mortality prompt us to focus on cancer patients to save lives. To better clarify the role of MPs and PS in coagulation of COVID-19 patients with cancer, we divide the disease into three major stages: early, intermediate and advanced stage.

### Pathological Mechanisms of Coagulation in COVID-19 Patients With Cancer

In the early stages of the disease, SARS-CoV-2 infects the epithelial cells of the upper respiratory tract and the conducting airways via angiotensin-converting enzyme 2 (ACE2) and type II transmembrane serine protease (TMPRSS2) receptors. Following binding to ACE2, TMPRSS2 cleaves and initiates receptor-binding spike protein, and mediates the fusion of the viral membrane with the membrane of host target cells. Once inside the target cells, the viral genome is then freed from the capsid for replication and translation. Cancer patients present with impaired innate immunity and dysregulated interferon response. Meanwhile, SARS-CoV-2 has evolved diverse mechanisms to evade host antiviral responses, which can prevent the signaling pathway of endogenous interferon induction. Therefore, COVID-19 patients with cancer manifest lower levels of interferon-I and interferon-III, moderate IFN-stimulated genes, and proinflammatory cytokine production, which indicates decreased antiviral immune function (Perico et al., 2021). Interestingly, evidence suggests that virus-inhibited interferon synthesis and high levels of proinflammatory cytokines are correlated with increased disease severity (Brain et al., 2021). This stage is characterized by mild symptoms, as only a small number of viruses have entered the bloodstream to activate the coagulation system. The body responds to the formation of blood clots by dissolving the partial microthrombi, causing elevated D-dimer levels in some patients. However, even with normal D-dimer levels, the possibility of thrombosis cannot be ruled out, since fibrinolytic function may not yet have started. Accordingly, we propose that cancer patients should be promptly treated with a therapeutic level of anticoagulation following COVID-19 diagnosis.

**FIGURE 1:**
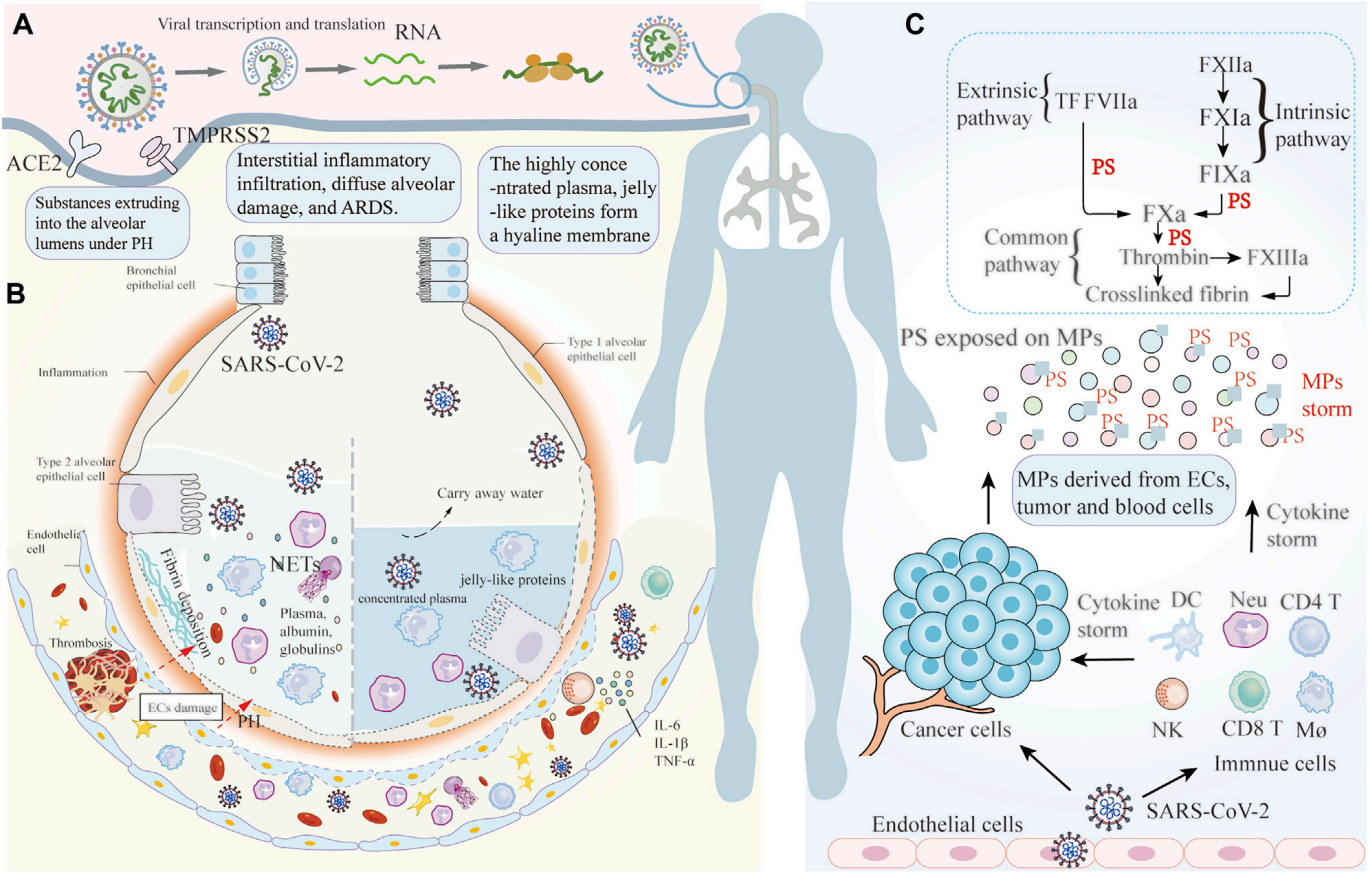
The pathophysiological mechanisms of pulmonary coagulation in COVID-19 patients with cancer. **(A)** SARS-CoV-2 infects the epithelial cells of the upper respiratory tract and the conducting airways via ACE2 and TMRPSS2 receptors. The virus enters into the host cell and is then freed from the capsid for transcription and translation. **(B)** Illustration of the mechanisms of thrombi formation in COVID-19 patients with cancer, combining a cancer-induced hypercoagulable state and procoagulant factors of COVID-19. Extensive microthrombi induce elevated pulmonary capillary pressure, eventually leading to PH. Virus infection promotes the damage to alveolar epithelial cells and ECs, causing substances within the blood vessel to extrude into alveolar lumens under the conditions of PH, which impedes the gas-exchange. NETs and activated immune cells extruding into the alveolar lumens are collectively responsible for interstitial inflammatory infiltration, diffuse alveolar damage, and ARDS. Mechanical ventilation can exacerbate the problem by drying the alveolar. The highly concentrated plasma and jelly-like proteins form a hyaline membrane in the alveolar space, and leading to ventilation-perfusion mismatch and serious hypoxia. **(C)** Massive activation of immune cells produces sustained inflammatory cytokines, which ultimately leads to cytokine storm. Cytokine storm further induces PS-positive MPs to be released from various cells, including blood and cancer cells, ultimately resulting in systemic thrombosis inside the vascular. ACE2: angiotensin-converting enzyme 2; TMPRSS2: type II transmembrane serine protease; PH: pulmonary hypertension; ECs: endothelial cells; ARDS: acute respiratory distress syndrome; RBCs: red blood cells; PLTs: platelets; Neu: neutrophil; NK cells: natural killer cells; DCs: dendritic Cells; Mø: macrophages; IL: interleukin; TNF Tumor necrosis factor-α: Tumor necrosis factor-α; IFN-γ; Interferon-γ; MPs: Microparticles; NETs: neutrophil extracellular traps.

In approximately 20% of infected patients, due to abnormal immune responses or high viral load or both, initial immune responses are insufficient to control viral replication (Leentjens et al., 2021). Consequently, the disease enters the intermediate stage, where the virus infects lung parenchyma along the airways and sustains exponential growth. Some patients rapidly progress to acute respiratory distress syndrome (ARDS) or even respiratory failure. The pathological characteristics of severe COVID-19 pneumonia include diffuse alveolar damage, hyaline membrane formation, epithelium and endothelium damage, and thrombosis in small and large blood vessels (Bridges et al., 2021). Several pathophysiological mechanisms have been proposed to explain the above manifestation. Tissue factor (TF) expressed on breast cancer tumor cells binds plasma factor VII/VIIa (FVII/FVIIa), forms TF/FVIIa complex, activates proteinase-activated receptor 2 (PAR-2), and then enhances coagulation by TF-positive MPs release. Furthermore, TF/FVIIa complex mediated PAR-2 activation also induces proangiogenic proteins and cytokines expression to shape the tumor microenvironment, and promotes the proliferation, invasion and migration of tumor cells (Featherby et al., 2019). P-selectin expressed on ECs enhances lung cancer-associated venous thromboembolism by recruitment of leukocytes from blood, making it a potential target to prevent thrombosis (Hisada and Mackman, 2017). Increased activity of plasminogen activator inhibitor 1 in pancreatic cancer breaks the equilibrium between coagulation and fibrinolysis, shifting towards the procoagulant side (Campello et al., 2019). In COVID-19, thrombo-inflammation is currently considered to be a key pathogenic factor in thrombosis, apart from direct ECs damage from virus infection. The virus activates the clotting cascade via multiple pathways, collectively promoting microvascular occlusion. ECs are crucial to maintaining blood fluidity, particularly in the microvasculature. ECs facilitate production of tissue factor pathway inhibitor (TFPI), thereby suppressing TF-driven factor X activation and thrombin generation. ECs also synthesize and release tissue-type plasminogen activator, which converts plasminogen into its active form, plasmin, and thus activates fibrinolysis and clot lysis (Leentjens et al., 2021). Virus-infected ECs lose their anti-thrombotic capacity through glycocalyx damage, surface expression of procoagulants, and apoptosis, thereby resulting in the exposure of the basement membrane and activation of the coagulation cascade. The virus can also activate PLTs and exacerbate the thrombo-inflammatory cascade. Activated PLTs bind to neutrophils, promote the generation of neutrophil extracellular trap (NETs), and initiate immunothrombosis (Yan et al., 2021). The mechanisms of thrombosis in COVID-19-infected cancer patients are not separated, but a result of the synergy between COVID-19 and cancer affecting the same coagulation system. Recently, the contribution of perivascular cells to coagulation has been recognized. Under the influence of oxidation, polyunsaturated fatty acids in the biofilms (especially, ω-6 or n-6 fatty acids, arachidonic acid, linoleic acid) become a target for oxidative stress-induced damage, and trigger lipid peroxidation (LPO) to generate reactive aldehyde, including the biologically most active, 4-hydroxynonenal (HNE). In the plasma of COVID-19 dead patients, protein adducts of HNE are relatively high compared to recovered patients. Immunohistochemistry showed abundant HNE-protein adducts, especially in the inflammatory edema and blood vessels of the lungs affected by COVID-19 (Žarković et al., 2021). In situations where there is endothelial cell damage, due to viral infection, hyperinflammatory cytokines, or abnormal complement activation, HNE can pass through damaged ECs and lead to the release of TF-positive MPs derived from perivascular cells (smooth muscle cells, pericytes, fibroblasts; Ansari et al., 2021). TF-positive MPs leak into the blood stream and come into contact with coagulation factors and induce coagulation within the vasculature. Extensive microthrombi results in elevated pulmonary capillary pressure, which eventually leads to pulmonary hypertension. Virus infection promotes necrosis and sloughing of alveolar epithelial cells and ECs. Pulmonary hypertension then causes the extrusion of blood vessel contents, including plasma, albumin, globulins, and even blood cells, into alveolar lumens, impeding gas exchange. Other substances extruded into the alveolar lumens including complement membrane attack complexes, NETs, and activated immune cells are collectively responsible for interstitial inflammatory infiltration, diffuse alveolar damage, and ARDS (Iba et al., 2021). The characteristics of immune signaling on the RNA-Seq to bronchoalveolar lavage fluid of COVID-19 show disrupted bronchoalveolar epithelial barrier, extensive immune infiltration, and hypercellular activity, which supports our ideas (Wang et al., 2021a). The alveolar type II cells can secret surfactants to allow efficient gas exchange, but severe inflammation, tissue damage, and the death of alveolar epithelial cells depress this process, so that the oxygen requirements of patients are barely met by increased vital capacity (Brain et al., 2021). The case fatality rate of the most severe patients is nearly 10%, and lung-protective mechanical ventilation is considered the most effective and efficient strategy to reduce multiple organ failure and mortality (Perico et al., 2021). As water is evaporated from the alveolar in patients undergoing mechanical ventilation, and the highly concentrated plasma and jelly-like proteins remain in the alveolar lumens, forming a hyaline membrane and ultimately leading to ventilation-perfusion mismatch and severe hypoxia. Hypoxia can induce vasoconstriction and enhance procoagulant phenotype, thereby contributing to vascular occlusion and exacerbating disease severity (Figure 1). Microthrombi formation in the pulmonary vasculature can explain the sudden progression to ARDS characterized by severe hypoxemia and lung edema (Yan et al., 2021). CT scan images of patients with deterioration show the accumulation of inflammatory exudates within the alveolar, low lung compliance, and increased lung weight.

In the advanced stages of the disease, the virus infects and bursts lung vascular ECs into the blood circulation. The virus activates innate immune cells via transmembrane pattern recognition receptors (PRRs). Once PRRs sense viral RNA, they will activate signaling cascades, including nuclear factor-κB (NF-kB) pathways, ultimately resulting in the expression of various cytokines and chemokines (Lowery et al., 2021). These inflammatory cytokines and chemokines subsequently recruit more innate immune cells (macrophage, neutrophil, dendritic, and NK cells) to produce sustained inflammatory cytokines, which is known as cytokine storm. Cancer has been linked to a pro-inflammatory state. Tumor cells modulate the recruitment of immune cells, particularly macrophages and T cells, which release proinflammatory cytokines, forming an inflammatory tumor microenvironment and aggravating cytokine storm in COVID-19 patients. Cytokine storm leads to the formation of MPs storm by inducing cellular apoptosis. In COVID-19 patients with cancer, additional factors increasing MPs generation include active release of extracellular vesicles by cancer cells, and tumor cell necrosis and apoptosis when the blood supply becomes inadequate to sustain tumor growth. A positive feedback loop of cytokines storm on the MPs storm promotes increasing microvascular coagulation, resulting in the area of microthrombi exceeding that of the damaged ECs and inadequate blood perfusion to the undamaged areas. Microthrombi formation, in turn, aggravates damage and apoptosis of blood cells, further facilitating MPs formation. These events promote a positive thrombo-inflammatory feedback loop, where hyperinflammation triggers a hypercoagulable state, leading to thrombosis inside the microvasculature (Figure 1). The laboratory indicators seen in COVID-19 patients include elevated fibrinogen, D-dimer, and inflammatory markers, with the levels of cytokines being associated with increased generation of fibrinogen. This is consistent with thrombo-inflammation, the combined effect of thrombosis and inflammatory processes, rather than typical diffuse intravascular coagulation (Iba et al., 2021; Sriram and Insel, 2021). Thrombosis induced by MPs storm leads to tissue damage, pathology progression, and a poor prognosis. Below, we focused on elucidating the mechanisms of MPs involvement in coagulation.

### Microparticles

MPs are extracellular vesicles (approximately 100–1000 nm in diameter) shed from the cell surface of healthy or damaged cells. In contrast, exosomes are smaller in diameter (approximately 40–100 nm) and released during the process of exocytosis (Zhang et al., 2018). Shedding of MPs from the cellular membranes is a tightly regulated and cytoskeleton-dependent process, that can be facilitated by activation of the apoptotic pathway, hypoxia, cytokine release or thrombin generation. Two distinct and well-characterized pathways have been identified, including cell activation and apoptosis. The shedding of MPs usually begins within a few minutes of the addition of related agonists, which work by increasing the cytosolic calcium concentration. In the process of apoptosis-dependent MPs formation, which commonly occurs over hours, dynamic membrane vesicles appear following cell shrinkage and DNA fragmentation. The mechanisms of MPs production involve the mobilization of intracellular calcium, kinases phosphorylation, and NF-κB activation (Nieri et al., 2016). MPs are released from all cell types, including blood cells (red blood cell, PLTs, macrophages/monocytes), vascular ECs, smooth muscle cells as well as tumor cells. There is ample evidence to support the role of MPs in the balance between coagulation and hemostasis (Tripisciano et al., 2017). Recent studies have also revealed that elevated levels of MPs are correlated with enhanced levels of coagulation activation biomarkers in diabetic retinopathy. The procoagulant MPs initiate and propagate coagulation in diabetes, which may account for the hypercoagulable state of diabetic retinopathy (Su et al., 2018). MPs derived from blood cells support enhanced thrombin generation potential, predominantly due to the rearrangement of plasma membrane phospholipids and PS externalization. PS exposure provides a catalytic surface for the attachment and interaction of coagulation factors on the MPs surface (Tripisciano et al., 2020). Recently, SARS-CoV2-ORF3A protein has been confirmed to induce apoptosis and increased level of PS that binds to annexin V (Ren et al., 2020). However, SARS-CoV2-ORF3A protein involvement in coagulation has yet to be fully studied. This is an interesting subject that has major implications for COVID-19 patients. In what follows, we focus on the elucidation of the mechanisms of PS exposure.

### PS Exposure on MPS Involved in Coagulation

**FIGURE 2:**
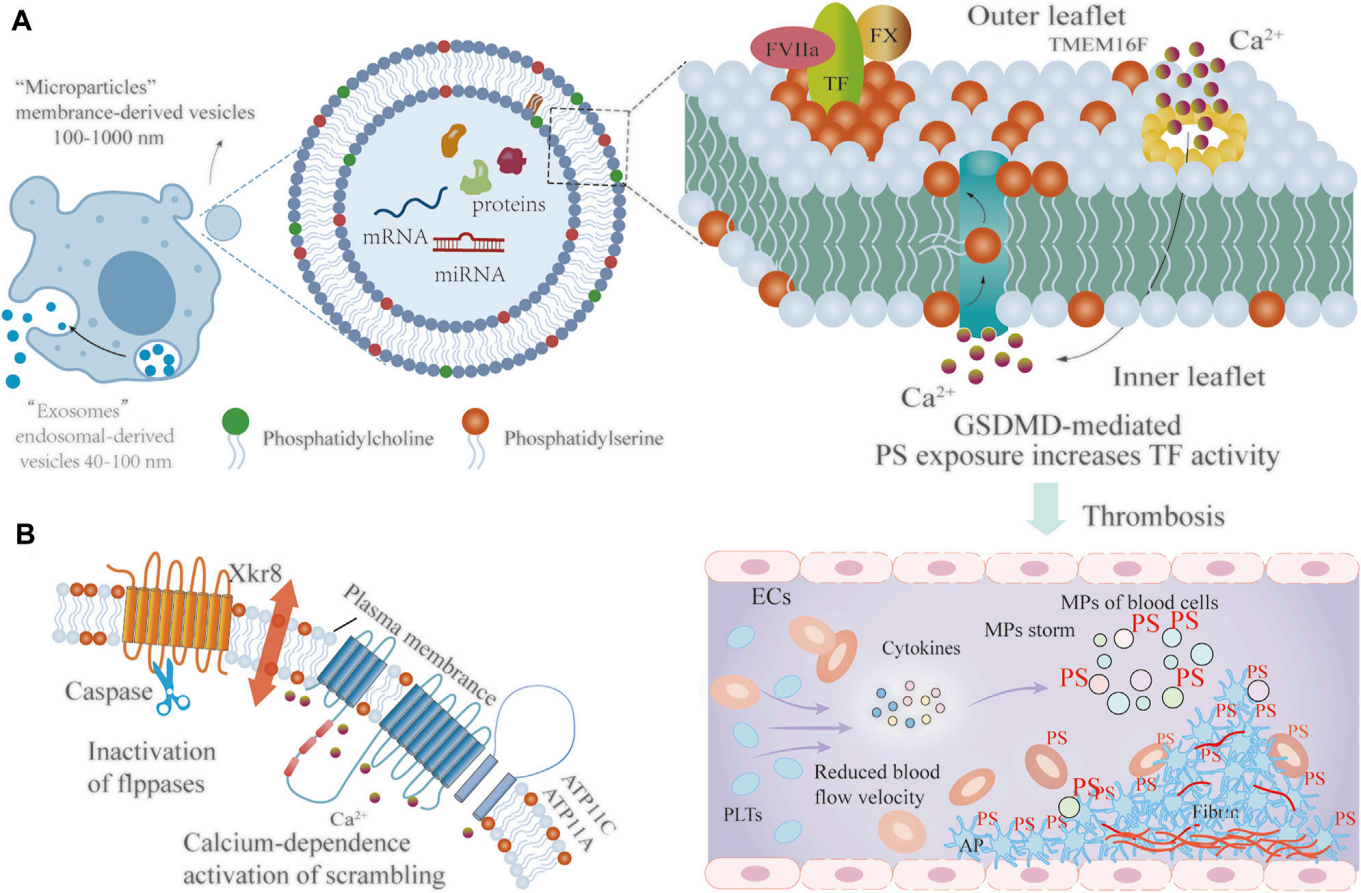
The mechanism of PS externalization involved in coagulation. **(A)** Extracellular vesicles can be classified into two types according to size: MPs (approximately 100–1000 nm) and exosomes (approximately 40–100 nm). GSDMD forms a pore, which leads to calcium influx, TMEM16F activation, and PS exposure, further enhancing the procoagulant activity of TF, and facilitating thrombosis **(B)** Owning to various stimuli, the caspase-dependent flippases ATP11A and ATP11C are inactivated while the calcium-dependent scramblase Xkr8 is activated, resulting in PS exposure on the outer membrane. FVIIa: Activated factor VII; TF: Tissue factor; FX: Factor X; PS: Phosphatidylserine; TMEM16F: Transmembrane protein 16F; ECs: Endothelial cells; PLTs: Platelets; AP: Activated platelets; RBCs: Red blood cells.; MPs: Microparticles; GSDMD: gasdermin D.

PS is an anionic phospholipid that provides a critical reactive surface for the coagulation cascade. Typically, PS is maintained in the inner layer of the cell membrane and rearranged on the outer face during activation and apoptosis (Yeung et al., 2008; Kang et al., 2019). The process of PS exposure on MPs is coordinated by flippases and scramblases and is irreversible. The type IV subfamily of P-type ATPases (P4-ATPase) are eukaryotic flippases, and transport lipids from the outer to the inner leaflet to maintain phospholipid asymmetry by ATP-dependent active transport. In normally growing cells, P4-ATPases, mainly ATP11A and ATP11C, are responsible for this process. Due to a variety of stimuli, calcium concentration within the cytoplasm increases, inactivating ATP11A and ATP11C, and therefore exposing PS on the outer membrane. Moreover, CDC50 family proteins, principally including CDC50A, are required for the folding and transport of multiple P4-ATPase. Deficiency of CDC50A leads to increased PS exposure on the outer cell membrane (Owens et al., 2011; Bevers and Williamson, 2016; Chang et al., 2020). Scramblase is another enzyme that facilitates the transport of phospholipids. Scramblases are commonly active in apoptotic cells or activated PLTs. Scramblases mediate non-specific, bi-directional, and ATP-independent phospholipid motion. Transmembrane protein 16F (TMEM16) and Xk-related protein 8 (Xkr8) are two scramblase proteins for PS transport. In cells undergoing apoptosis, Xkr8 is cleaved by active Caspase 3 or 7, and subsequently, a cleaved form of Xkr8 in complex with basigin or neuroplastin induces the activity of scramblases. Xkr8-mediated PS exposure is slow and irreversible, thereby promoting phagocytosis of apoptotic cells. Together, caspase-dependent Xkr8 activation and calcium-dependence inactivation of P4-ATPase result in sustained PS exposure on MPs (Shin and Takatsu, 2020; Ito et al., 2021). TMEM16F is a calcium-dependent phospholipid scramblase, that mediates PS exposure on MPs released by activated PLTs. Recent studies have revealed that caspase-11-gasdermin D forms the pore, which causes calcium influx, TMEM16F activation, and PS exposure, further enhancing the procoagulant activity of TF, and facilitating the blood coagulation cascade (Yang et al., 2019; Stark and Massberg, 2021; Figure 2).

### The Mechanisms of PS Storm Involved in Coagulation

In the context of proinflammatory cytokines released by COVID-19 and the inflammatory microenvironment in cancer, the amount of PS exposure on MPs released by apoptotic cells is massive enough to form a PS storm. PS exerts an essential role in blood coagulation initiation, amplification, and propagation. At the initiation step, TF expression on damaged monocytes increases, but as TF exists in an encrypted state, even combination with activated FVIIa does not exert significant procoagulant effects (Wang et al., 2019). However, PS exposure is a critical factor of TF decryption to initiate the extrinsic pathway of coagulation. Furthermore, PS in combination with TF/FVII complex contributes to steric stabilization of catalytic site and enhances PS-mediated TF procoagulant activity. Apart from PS, viral infection can also activate acid sphingomyelinase (ASMase), which hydrolyzes of sphingomyelin leading to TF decryption. A recent study showed that infection of human monocyte-derived macrophages with SARS-CoV-2 spike protein pseudovirus resulted in enhanced TF procoagulant activity and release TF + MPs through this pathway. This process neither affects the re-synthesis of TF nor depends on PS externalization (Wang et al., 2021b). TF/FVIIa complex further cleaves FIX and FX into FIXa and FXa. FXa assembles with FVa on the surface of monocytes, and the resulting prothrombinase complex generates thrombin through proteolytic cleavage of prothrombin in blood plasma. The relative concentration of TF/FVIIa complex and TFPI determine the duration of this initial phase. The generated FXa binding to TFPI forms the quaternary complex with TF and FVIIa, thereby inhibiting FVIIa and coagulation. Subsequently, FIXa is moved from the surface of monocytes to the activated platelet surface at the site of damage (De et al., 2013).

During the amplification phase, low concentrations of thrombin activate PLTs adhering to the site of injury, thus inducing the release of FV and FVa from α-granules. Thrombin activates FV in circulation, and induces activation and release of FVIII from von Willebrand factor (vWF). Moreover, small amounts of thrombin activate FXI into FXIa to enhance the concentrations of active coagulation factors. In this phase, FVIIIa and FVa in combination with phospholipids on the PLTs surface do not require calcium ions, but only the presence of PS (De et al., 2013). Activated PLTs also participate in the coagulation process. Following ECs activation induced by the virus, increased P-selectin and vWF expression initiate platelet adhesion to the endothelium through binding with PLTs P-selectin glycoprotein ligand-1 and glycoprotein (GP)Ib/IX/V complex. Intercellular adhesion molecule-1 promotes PLTs adhesion via the binding of fibrinogen with GPIIb/IIIa on the PLTs membrane. Moreover, exposed subendothelial collagen facilitates PLTs adhesion and activation by binding to GP VI, eventually resulting in PLTs aggregation (Jansen et al., 2018), triggered by fibrinogen binding to active integrin αIIbβ3. Activated PLTs release mediators, especially ADP and Thromboxane A2, which attract circulating PLTs to the growing thrombus (Mackman et al., 2020).

PLTs promote the blood coagulation cascade through PS exposure and the release of clotting mediators, including TF, polyphosphate. PLTs activate FXII by MPs formation and polyphosphate release, and increase the generation of thrombin and fibrin (Müller et al., 2009). Polyphosphate -dependent FXII activation does not accelerate clot formation, but increases the stability of fibrin clots. Activated PLTs can also trigger the extrinsic coagulation pathway. Studies suggest that PLTs cannot directly secrete TF, but CD40L expression on PLTs can induce TF expression on monocytes, further activating the extrinsic coagulation cascade (Labberton et al., 2016).

**FIGURE 3:**
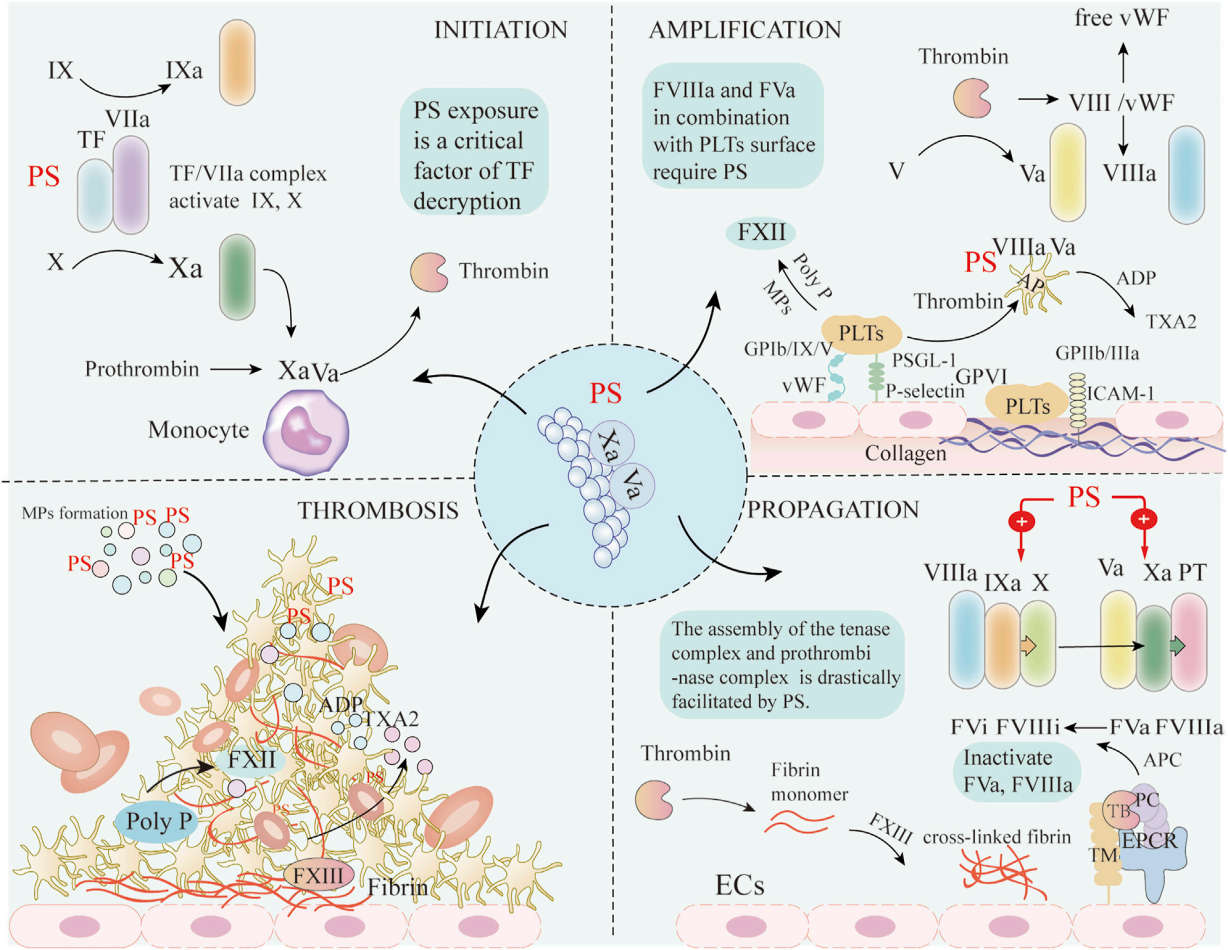
The mechanisms of PS involved in coagulation. During the initiation step of the coagulation cascade, PS exposure mediates TF decryption initiating the extrinsic coagulation pathway. TF/FVIIa complex can then activate FIX and FX. Subsequently, FXa assembles with FVa on the surface of monocytes, and the prothrombinase complex generates thrombin by cleavage of prothrombin in blood plasma. During the amplification phase, thrombin activates circulating FV and FVIII, causing its release from vWF. Subsequently, FVIIIa and FVa combine with PS-rich phospholipids on the activated PLTs surface. Following ECs activation, increased P-selectin and vWF expression initiate PLTs adhesion to the endothelium through binding with PLTs PSGL-1 and glycoprotein GP Ib/IX/V complex, respectively. ICAM-1 promotes PLTs adhesion via binding of fibrinogen with GPIIb/IIIa on the PLTs membrane. Moreover, exposed subendothelial collagen promotes PLTs adhesion and activation by binding to GPVI, eventually leading to PLTs aggregation. Activated PLTs release mediators, especially ADP and TXA2, which attract circulating platelets to the growing thrombus. PLTs activate FXII by MPs formation and poly P release, and increase the generation of fibrin. In the propagation phase, exposed PS facilitates the assembly of the tenase and prothrombinase complexes of the coagulation cascade. Thrombin cleaves soluble fibrinogen into fibrin and activates FXIII, which forms cross-linked and stable fibrin. The above coagulation processes ultimately lead to platelet activation and thrombus formation. ECs: Endothelial cells; PS: Phosphatidylserine; TF: Tissue factor; VIIa: Activated factor VII; Xa: Activated factor X; Va: Activated factor V; VIIIa: Activated factor VIII; PT: Prothrombin; TXA2: Thromboxane A2; ICAM-1: Intercellular adhesion molecule 1, PLTs: Platelets; AP: Activated platelets; vWF: von Willebrand factor; GP: Glycoprotein; Poly-P: polyphosphate; FXII: Factor XII; FXIII: Factor XIII; TB: Thrombin; PC: Protein C; APC: Activated protein C; TM: Thrombomodulin; ERCP: endothelial protein C receptor.

The propagation phase consists of two primary processes. FVIIIa assembles with FIXa to generate active tenase complexes that activates FX. Subsequently, FXa binds to FVa to form the prothrombinase complex, which is catalyzed by the PS-rich lipid membrane surface, leading to a burst of thrombin formation, which is required for rapid and local fibrin mesh formation. PS is involved in the amplification phase by binding two types of protein domains: γ-carboxyglutamate-rich (Gla) domains (prothrombin, FVII, FIX, and FX) and C-type lectin domains (FV and FVIII) (Gilbert et al., 2015; Liu et al., 2019). Specifically, the assembly of the tenase (factors VIIIa, IXa) and prothrombinase (factors Va, Xa) complexes drive the coagulation cascade. Ca^2+^ functions as a bridge between negatively charged domains of coagulation factors and PS on the MPs surface (Tripisciano et al., 2020). Neutralizing exposed PS with lactadherin, a PS-binding protein, significantly inhibits the production of thrombin and fibrin. Thrombomodulin on the surface of ECs, in combination with thrombin complexes with endothelial cell protein C receptor, converts protein C to activated protein C. Activated protein C, together with its cofactor protein S, can inactivate FVa and FVIIIa by proteolytic cleavage, thereby exerting a negative regulatory role on thrombin, and avoiding excessive thrombin production (Sriram and Insel, 2021). Thrombin cleaves soluble fibrinogen into fibrin monomers, which then polymerize to form the fibrin strands. PLTs then aggregate together to stabilize the platelet plug and activate FXIII, which forms cross-linked and stable fibrin (Nieswandt and Watson, 2003; Figure 3).

### Therapeutic Strategies

Effective anti-cancer therapies are necessary for COVID-19 patients with cancer. However, in addition, alternative therapeutic approaches to ameliorate symptoms and delay disease progression should be used. A prethrombotic state of cancer, together with extensive ECs damage and activation, along with MPs storm and PS storm collectively aggravate systemic thrombosis. Effective anticoagulant therapeutics are therefore highly warranted. Anticoagulation remains the cornerstone for the prevention and treatment of venous thromboembolism. Given the prominent role of PS in COVID-19-associated coagulation, neutralizing PS is necessary for patient treatment. Our previous studies have compared lactadherin with annexin V, and the advantages of lactadherin are as discussed below. First, lactadherin has greater sensitivity and specificity, allowing PS exposure to be detected on the outer leaflet earlier. Second, annexin Ⅴ selectively binds to concave cellular membrane surfaces with the degree of binding to PS having an inverse relationship with membrane curvature. In contrast, lactadherin binds to PS in the absence of either calcium ions or PE, and binding is positively correlated with cell membrane curvature. Third, lactadherin is a more potent and highly efficient inhibitor of coagulation factors. Lactadherin suppresses thrombus formation by competing effectively for FV and FVIII binding sites (Shi et al., 2008). Compared to healthy volunteers and septic ICU patients, PS externalization in serum from COVID-19 ICU patients is dramatically increased. Enhanced serum PS externalization of COVID-19 ICU patients correlates with increased sequential organ failure assessment score. More importantly, PS externalization in patients with thrombosis is higher than those without. This evidence shows that PS externalization may exert an essential role in coagulation of COVID-19 patients, and neutralizing exposed PS could significantly improve hemostatic balance (Althaus et al., 2021; Table 1).

**TABLE 1:** Treatment strategies for COVID-19 patients with cancer.

Treatment	Drugs	Significance	References
Neutralizing exposed PS	Lactadherin	PS transport to the outer leaflet of the plasma membrane of the infected alveolar and ECs is one of the underlying mechanisms in the coagulation abnormalities of COVID-19. Lactadherin can improve the coagulation of patients	Argañaraz et al. (2020)
FXII inhibitors	3F7	The inhibition of FXII as means to inhibit the thromboinflammatory response incited by severe COVID-19 appears an attractive and rational therapeutic target	McFadyen et al. (2020)
FXI inhibitors	IONIS416858	Prophylactic FXI inhibition has been predicted to be beneficial in VTE, thrombosis in end-stage renal disease, and for anticoagulation in patients undergoing extracorporeal membrane oxygenation or hemodialysis	Carle et al. (2021)
Thrombin inhibitors	Dabigatran	Dabigatran is the first DOAC that has a direct reversible inhibitory effect on thrombin, which may be an alternative drug	Shnayder et al. (2021)
FXa inhibitors	Rivaroxaban	DOAC therapy compared to VKA therapy at the time of COVID-19 diagnosis demonstrates lower risk of arterial or venous thrombotic outcomes, without increasing the risk of bleeding	Rivera-Caravaca et al. (2021)
Anticoagulant	LMWH	Reports have suggested that LMWH treatment reduces mortality in COVID-19 patients with an elevated D-dimer or elevated sepsis-induced coagulopathy score	McFadyen et al. (2020)
Antiplatelet therapy	Aspirin	The use of aspirin during hospitalization for COVID-19 could be associated with lower mortality risk and shorter duration of mechanical ventilation, without increased risk of bleeding	Santoro et al. (2021)
	Clopidogrel	Antiplatelet therapy, including clopidogrel, might be effective in improving the ventilation/perfusion ratio in COVID-19 patients with severe respiratory failure	Santoro et al. (2021)
Anti-inflammatory treatment	Tocilizumab	Tocilizumab is associated with lower mortality and other clinically relevant outcomes in hospitalized patients with moderate-to-critical COVID-19	Kyriakopoulos et al. (2021)
	Anakinra	Treatment with anakinra reduces both the need for invasive mechanical ventilation and mortality risk of hospitalized non-intubated patients with COVID-19 without increasing the risk of adverse events	Barkas et al. (2021)
Immunomodulation	Corticosteroids	Moderate-certainty evidence shows that systemic corticosteroids probably slightly reduce all-cause mortality in people hospitalized because of symptomatic COVID-19	Wagner et al. (2021)
Vaccine	—	The currently approved vaccines have been extremely effective in preventing COVID-19, particularly severe disease. However, viral mutations compromises vaccine efficacy	Tregoning et al. (2021)

Abbreviations: PS, phosphatidylserine; ECs, endothelial cells; FXII, factor XII; FXI, factor XI; FXa, activated factor X; ALMWH, low molecular weight heparin.

FXII is responsible for activation of the contact pathway. FXII and FXI are important for relatively early initiation of the coagulation cascade. Inhibition of coagulation through these upstream mediators is more effective than inhibition of downstream coagulation factors such as FX and thrombin. FXII and FXI inhibitors suppress coagulation, but also decrease the consumption of other coagulation blood factors and reduce the incidence of disseminated intravascular coagulation (Bonaventura et al., 2021). Additionally, patients with congenital FXI and FXII deficiency present with slight or no bleeding. Therefore, FXII and FXI inhibitors have the potential to combine efficacy and safety. FXI inhibitors include antisense oligonucleotides, monoclonal antibodies, aptamers, and small molecules. Drugs currently under investigation include IONIS416858, bay1213790, MAA868, among others. The most advanced group of FXII inhibitors are fully human or humanized monoclonal antibodies such as 3F7. FXII inhibitors are considered to be an attractive therapeutic target in severe COVID-19 (Table 1).

Direct-acting oral anticoagulants (DOACs) bind to the active site of the target enzymes and block their activity. DOACs include thrombin inhibitors (dabigatran) and factor Xa inhibitors (rivaroxaban, apixaban or edoxaban). Predictable anticoagulant effects enable these drugs to be administered at a fixed dose, with no dose adjustment or laboratory monitoring (Ay et al., 2019). DOACs are reportedly an effective treatment option for patients with cancer and acute venous thromboembolism (VTE) because they inhibit cancer-induced prothrombotic state and attenuate COVID-19-related VTE. Moreover, compared to vitamin K antagonist therapy, DOACs produce lower thrombotic risk and no increased risk of bleeding in COVID-19 patients. Clinical guidelines recommend low-molecular- weight heparin (LMWH) as the first-line treatment of short- and long-term cancer-related VTE. The significant advantages of LMWH are rapid onset through intravenous administration and good reversibility. LMWH exerts anticoagulant effects by multiple mechanisms. LMWH can bind to and induce a conformational change in antithrombin III (ATIII), leading to a 1000-fold increase in its ability to suppress thrombin, FXa, and other coagulation serine-proteases. It has been demonstrated that LMWH is associated with reduced mortality in COVID-19 patients with elevated D-dimer levels (Table 1).

PLTs are implicated in the pathogenesis of venous thrombosis (Qiu et al., 2021). Evidence of PLTs activation in patients with acute VTE demonstrates that PLTs play an essential role in the amplification and propagation of venous thrombosis. Recent trials have suggested that aspirin is effective in primary and secondary prevention of thromboembolic events (Chan and Weitz, 2019). COVID-19 hypoxia, ECs damage, and oxidative stress result in excessive PLTs activation and apoptosis through effects on the metabolism and function of PLTs mitochondria, promoting platelet-rich thrombotic microangiopathy. Increased PLTs activation (measured through expression of CD63 and P-selectin) is linked to a poorer prognosis. However, aspirin can significantly reduce the risk of mechanical ventilation, intensive care unit admissions, and in-hospital mortality. Therefore, substantial numbers of COVID-19 patients could benefit from treatment with aspirin. Clopidogrel, another alternative therapy, antagonizes the P2Y12 receptor, suppressing PLTs activation and improving the coagulation state (Sriram and Insel, 2021). Furthermore, clopidogrel improves the ventilation/perfusion ratio in COVID-19 patients with severe respiratory failure (Table 1).

Early prophylactic anticoagulant therapy has important implications for patient prognosis. First, early antithrombotic therapy to maintain blood flow patency contributes to viral clearance, reduces cell apoptosis, attenuating the formation of cytokines and MPs storm, maintaining oxygen saturation, and avoiding disease progression. Second, early antithrombotic therapy can inhibit activation and aggregation of blood cells, maintain air-blood exchange and the integrity of pulmonary circulation, avoiding microthrombi, and subsequent pulmonary hypertension, and averting alveolar plasma leakage and systemic circulation hypoxemia. Third, early therapeutic interventions to suppress thrombosis can reduce the severity of the disease, and prevent organ injury and the need for ventilator support. For diseases involving extensive ECs damage, massive release of proinflammatory cytokines characterized by excessive immune activation, and the apoptosis of blood cells, anticoagulant treatment should be performed as early as possible before the emergence of critical illness and the establishment of irreversible vascular pathology. Early intervention has to potential to improve patient coagulation, inhibit the progression of the disease and decrease mortality (Kollias et al., 2021).

The intermediate stage manifests as a hypercoagulable tendency, and continuation of anticoagulant therapy should be recommended. The dose of anticoagulant therapy should be adjusted to be higher than at the early stage, although the efficacy is not as favorable at this later stage. Accumulating evidence has indicated the effects of anticoagulant therapy in advanced COVID-19 are not satisfactory. Clinical outcomes of anticoagulation in COVID-19 patients with cancer may depend on the timing of initiation and disease process, and be implicated in disease severity as well as inflammatory and clotting state at the beginning of treatment. Although several organ systems exhibit significant coagulation activation in severe COVID-19 patients, the initiation of treatment may be after the occurrence of severe COVID-19, therapeutic dose of anticoagulant therapy may be too late to change the consequences of the established disease process. At this time, inflammation and the coagulation cascade amplify each other, to form extensive MPs, PS, and coagulation storm. Therefore, even though anti-inflammatory and anticoagulant therapeutics may remove the initiating factors, the amplified response still remains. Thrombocytopenia, platelet dysfunction, and deficiency of clotting factors promote bleeding complications, which commonly emerge in critically ill advanced COVID-19 patients. Depleted blood cells and coagulation factors shift the hemostatic balance towards the bleeding side, such that the antithrombotic therapy is not only ineffective but aggravates bleeding (Gu et al., 2021). According to recent guidelines, the prevention of thromboembolic complications is needed for severe COVID-19 illness even after discharge. Patients with a high risk of VTE and low bleeding risk after discharge are advised to continue thromboprophylaxis with LMWH or DOACs (Ortega-Paz et al., 2021).

### Anti-Inflammatory Therapy

Cytokine storm is the trigger of MPs storm, which makes patients more prone to thrombotic events. Inflammatory signaling pathway antagonists and anticoagulant therapy may be more effective than either medicine alone in the prevention of thrombosis and death. In the early stage, inflammation recruits immune cells to the infected sites, activates their protective functions, and promotes the development of adaptive immunity. Therefore, premature intervention may disrupt the formation of effective immunity. Subsequently, feed-forward mechanisms take over, so that explosive inflammatory amplification and its hazardous consequences cannot be controlled by intervention. Therefore, the optimal timing for anti-inflammatory therapy may be at the first signs of respiratory distress (e.g., a slight decline in blood oxygenation level, mild dyspnea, chest X-ray images showing early signs of pneumonia). Remarkably, IL-6 is a critical mediator of multiorgan dysfunction, including acute kidney injury. A recent meta-analysis reported that IL-6 is associated with poor clinical outcomes (Legrand et al., 2021). Consequently, IL-6 receptor antagonist tocilizumab is a potential treatment option to limit multiple organ failure and disease severity. IL-1β receptor antagonist anakinra has also been shown to reduce mortality (Kyriazopoulou et al., 2021). In COVID-19 patients, dexamethasone is only effective in some patients requiring mechanical ventilation and oxygen supplementation (Marconi et al., 2021; Table 1). Because cancer-mediated immune suppression coupled with dexamethasone inhibits the production of effective antibodies, delaying viral clearance and aggravating disease progression, dexamethasone is not recommended in COVID-19 patients with cancer.

### Other Therapeutic Approaches

Despite the availability of numerous vaccines against SARS-CoV-2, the morbidity and mortality of COVID-19 remain high. Furthermore, the continuous evolution of variants raises concerns for existing vaccine efficacy (Lowery et al., 2021; Zhou et al., 2021; Table 1). Certain patient populations, including cancer and immunosuppressed patients, may be unable to produce an effective antibody response, therefore, we urgently need to adopt appropriate treatment to improve patient prognosis.

## Conclusion

COVID-19 pandemic poses unprecedented challenges to cancer patients worldwide. Increasing insights into the pathophysiological mechanism of SARS-CoV-2 infection and how it leads to critical illness and thrombotic complications will hopefully lead to the development of new treatment methods to reduce acute and long-term sequelae. This review, by examining an extensive literature in combination with the results of post-mortem autopsy, has summarized the possible mechanism of lung microvascular coagulation and systemic thrombosis in COVID-19 patients with cancer and explored the effects of cytokine, MPs, and PS storms on systemic coagulation abnormalities. We have highlighted the role of PS in coagulation initiation and amplification. We have also discussed the most promising treatment interventions to limit disease progression in the early stage, mainly anticoagulant therapy, enumerated the existing therapeutic agents repurposed to treat COVID-19 patients with cancer, and identified the optimal timing to maximize the efficacy of these drugs. We elaborated on the significance of early antithrombotic therapy. We also provide evidence that anticoagulant treatment should be carried out as soon as possible to reduce the mortality and the risk of sequelae, whenever patients present with extensive ECs damage, high levels of cytokines, and MPs storm. Due to the increased risk of thrombosis in cancer patients and the potential for coagulation disturbances in the pathophysiological mechanisms of many diseases, it is likely that early anticoagulant treatment will continue to be important for this patient population in response to future epidemics and pandemics.
